# Parenting during the COVID-19 Lockdown in Portugal: Changes in Daily Routines, Co-Parenting Relationships, Emotional Experiences, and Support Networks

**DOI:** 10.3390/children8121124

**Published:** 2021-12-03

**Authors:** Ana P. Antunes, Silvana Martins, Laura Magalhães, Ana T. Almeida

**Affiliations:** 1 Department of Psychology, Faculty of Arts and Humanities, University of Madeira, Campus Universitário da Penteada, 9020-105 Funchal, Portugal; aantunes@uma.pt; 2 Research Centre on Child Studies, Institute of Education, University of Minho, Campus de Gualtar, 4710-057 Braga, Portugal; monteiro.laurapatricia@gmail.com; 3 Health Sciences Research Unit: Nursing, Nursing School of Coimbra, Avenida Bissaya Barreto, Polo C, 3046-851 Coimbra, Portugal; b12012@ese.uminho.pt

**Keywords:** parenting, daily routines, co-parenting relationship, emotional experience, family support networks, family resilience, positive parenting, COVID-19 lockdown, mixed-method research

## Abstract

The COVID-19 pandemic challenged parental resources pertinent to coping with lockdowns. The main objective of this work was to study parenting during the COVID-19 lockdown. Specifically at focus were parental behaviors concerning key domains for the family (daily routine, co-parenting, emotional experience, and support network) and changes related to the pandemic and associated with the parents’ employment statuses. An online survey was carried out through an ad hoc questionnaire where participants completed questions about their sociodemographic data and rated how much their family routines, their co-parenting relationship, their emotional experiences, and the support available in the family network varied on a 5-point scale. The participants included 1384 parents, of which 286 responded to open questions regarding impactful experiences during the lockdown. The results showed differences in daily routine, co-parenting, emotional experience, and support network according to the parents’ employment statuses. Between-group comparisons showed that at-home parents caring for children with governmental aids generally revealed more positive parenting behavior changes, while at-home parents who were teleworking reported more difficulties in parent-child activities and co-parenting. Furthermore, the content analysis of the data confirmed how important themes such as family dynamics, professional activities, and the relationship with the school community were throughout the participants’ accounts of gains and losses. Overall, parents’ employment statuses are associated with diverse experiences during lockdown. The COVID-19 pandemic highlighted the importance of family resources and parental resilience, particularly during circumstances jeopardizing the ever-sensitive work-family balance.

## 1. Introduction

In March 2020, after rapid escalation of the COVID-19 pandemic, the World Health Organization [1] declared it a global pandemic. In the context of this pandemic, governments around the world have taken restrictive measures to prevent and control contagions by COVID-19. The Portuguese government formally declared a state of emergency on 18 March and ordered a lockdown [2]. Only in May was that lockdown measure changed and some activities recommenced. During this period, parents and children stayed at home and found themselves sharing family routines, professional tasks, child care, and school activities. All of a sudden, parents had to respond to professional demands while trying to meet children’s needs (cognitive, emotional, and physical), to adjust their schedules and spaces, and to assume a more prominent role in the formal education of their children while aiming to control the impact of distance teaching [3,4].

Globally, confinement measures (e.g., obligations to stay at home, severe restrictions on movements, disruption in routines, and physical distancing) increased parents’ pressures, stress, and anxiety and demanded additional resources to help preserve children and families’ mental health. Rather timely evidence from a systematic review concerning the psychological effects of compulsory lockdowns during previous epidemics (for example, Ebola, H1N1, MERS, and SARS) [5] concluded that people who completed a quarantine period were more likely to have suffered psychologically compared with people who were not in quarantine. Additionally, this review reported a higher prevalence of psychological symptoms, namely, post-traumatic and depressive symptoms, stress, and anxiety, in people who experienced quarantines [5]. Regarding the COVID-19 pandemic, the results of already available studies point out distinct outcomes to families worldwide. Among the outcomes are, for instance, those indicating that some parents had high levels of psychological distress and presented several difficulties [6] and that the impact of the confinement on children was mediated by its impact on parents: when parents faced more difficulties dealing with the lockdown and experienced more stress, children’s problems increased [7]. In fact, several aspects of family dynamics were challenged, to which the responses were diverse. Humor and well-being were seemingly affected, revealing that anxiety and depressive symptoms were more frequent in children whose parents experienced higher stress [8].

During the lockdown, remote work was a compulsory measure that was not planned and, in some cases, lasted for months. Consequently, “for working parents and carers, the school closures, and the closing of other care facilities have made working from home challenging. (…) These workers find balancing their work and care responsibilities challenging and are experiencing new dynamics in managing their work-life balance.” [9] (p. 3). Particular circumstances of the COVID-19 lockdown presented unforeseen challenges to families’ functioning. At-home education during the COVID-19 lockdown was also a novelty demanding parents and children to cope. Some children felt at-home education exhausting due to excessive schoolwork [10] and, again, their well-being was associated with parents’ psychological well-being [11]. Concretely, parents who met the criteria for major or severe depression or parental stress did not perceive themselves as well prepared to educate their kids at home. Additionally, children from parents with moderate or severe anxiety showed higher anxiety levels. Additionally, in general, higher anxiety scores were associated with parental stress [11].

In addition, the lockdown effects on a couple’s relationship dynamics and the need for more equal division of tasks were noted [12] and the worsening of socioeconomic inequalities was highlighted [13]. Furthermore, changes in the parent’s work situations are referenced as an aspect that constrains the family members’ well-being. Undoubtedly, the advantages of working from home can be highlighted (diminished time, stress, and costs with regard to transportation; improved work efficiency; and greater work control) as well as disadvantages (home office constraints, work uncertainties, inadequate tools, and a heightened overall workload) [14]. Nevertheless, the COVID-19 pandemic forced several workers to stay home and work remotely. In that sense, the International Labour Organization (ILO) constructed a practical guide so employees and employers could handle not only this new situation but telework under normal conditions more effectively [9].

Nevertheless, despite the difficulties experienced by parents and the changes in family dynamics, which might have caused a sudden increase in emotional tension, the lockdown did not alter the affective relationship and the warmth and caring behavior of parents with their children, as stated in a Dutch study [15]. Indeed, an affective and supportive network seemed crucial to both parents and children. A Finnish study stressed how schools and teachers acted as important support networks for family resilience in the abrupt change toward remote learning [16]. In fact, adaptation or maladaptation of each member or family group was mediated by family processes [17]. Considering a systemic perspective, the different ways of dealing with highly stressful experiences and social environments must be interpreted considering the vulnerability, risk, and resilience of families [18,19,20,21]. Changes associated with the lockdown restrictions may account for an increase in anxious symptoms and higher levels of stress, which negatively influence family relationships. Burdens and limitations challenged the parents’ control and satisfaction of personal needs. Nonetheless, other families saw this period as an opportunity to engage with their children in play and learning and to support and help their children coping with the uncertainty and the novelty [22,23,24]. Therefore, individual and collective experiences related to the lockdown are dynamic, multifaceted, and complex and should not be only framed as negative, as sometimes implicit in the scientific literature on quarantines [24]. To some, the positive parenting roles enacted during this adverse period allowed the family to be creative, to solve their problems, and to persevere [17].

In conclusion, the research related to the impact of the pandemic on parenting does not gather a consensus. Some studies referred to parents who experienced situations of stress, anxiety, and conflict [12,25] as evidence of an increasing risk of maltreatment [26,27]. In turn, some studies associated the lockdown with an opportunity for good family coexistence, in which parents recognize the positive effects on themselves and their children [24,28].

In this sense, identifying the parents’ resources and responses to coping during the lockdown may foster awareness of the opportunities and challenges to strengthen parental capacities and family resilience. Therefore, given the unprecedented situation, the main objectives of this study were (a) to explore parents’ behaviors in key areas of family functioning (daily routine, co-parenting, emotional experience, and support network) in a sample of Portuguese parents during COVID-19 lockdowns and (b) to analyze changes in parents’ behaviors (stayed the same, decreased, and augmented) across daily routines, co-parenting, emotional experience, and support network) according to the parents’ employment statuses (at-home parents with children under 12 with a licensed work leave and governmental aids, at-home parents who were teleworking, parents working out-of-home as usual, and parents who were unemployed) associated with the family being confined at home. Given the exploratory nature of the present study, we envisioned, first, to examine the pattern of changes in the abovementioned key areas of parents’ functioning from a quantitative approach and, second, to complement this data set with a qualitative analysis of the parents’ responses to an open-end question on most impactful experiences to family functioning during the COVID-19 lockdown.

## 2. Method and Materials

### 2.1. Study Design

In this study, we used a mixed-methods approach [29,30] to account for the quantitative data (scale dimensions) and qualitative data (participants’ open-ended responses). First, we performed a set of descriptive analyses to ascertain the distribution of responses across the four parenting dimensions (daily routines, co-parenting, emotional experience, and support network). Second, one-sample t-tests were conducted to explore the differences between the scales and the subscales and the mid-point response that indicates no change, followed by a series of one-way analyses of variance (ANOVAs) to ascertain differences related to parents’ work statuses in the four abovementioned parenting dimensions. Multiple post hoc comparisons were accomplished using Bonferroni test controlling for type I error rate per comparison and the Bonferroni correction. The inferential analysis allowed us to better understand the pattern of changes in the participants’ ratings on several parenting dimensions evaluated on the scales. A qualitative analysis of the short narratives (a-few-word sentences to several paragraphs) used basic analytic processes to code and categorize the data gathered on the written responses, identifying patterns and regularities and, subsequently, abstracting conceptual meaning out of the clustered codes [31]. Despite not all participants responding to the open question, the subjective testimonies about the most impactful experiences during the lockdown allowed for an extensive picture of the changes registered in some families. To accommodate for the contribution from quantitative and qualitative data, we used a concurrent embedded strategy as defined by Creswell: “a concurrent embedded approach has a primary method that guides the project and a secondary database that provides a supporting role in the procedures. Given less priority, the secondary method (quantitative or qualitative) is embedded, or nested, within the predominant method (qualitative or quantitative)” [32] (p. 214). Therefore, quantitative and qualitative data were concurrently collected (they were collected at the same stage of the study) and the two databases (quantitative data from the scales, the predominant method, and qualitative data from the open question, the secondary method) were compared to determine the degree of convergence or discrepancy, to integrate them, as well as to gain a wider perspective of the changes in families that the secondary method could bring.

### 2.2. Participants

The participants included 1384 parents (89.9% mothers), recruited through Facebook, WhatsApp, and other social media platforms. The inclusion criteria were being a parent or substitute caregiver of one or more children 18 or under, living at home.

The participants were predominantly of Portuguese nationality (96.6%) and ranged in age from 20–62 years old (M = 38.92, SD = 6287). Most of them were married or had a stable relationship (81.5%), had a university degree (71.9%), and were employed (82.7%), and of these, 42.8% of parents were teleworking. Still, 9.7% of parents were unemployed, and 23.3% benefitted from governmental aids to support families in their caregiving for children under 12.

For the number of children living at home, the parents in this sample had between one and five children, with most of these families having one (43%) or two (45%) children. The age of the children varied between 0–18 years old (M = 6.51, SD = 4.47) and had no special educational needs (94%).

On average, these families experienced up to 58.68 days in lockdown, lived in apartments (54.6%), and had terraces or open-air spaces (69.6%).

### 2.3. Measures

Despite previously acknowledged measures on parenting (e.g., Me as a Parent) [33,34], the authors’ goal was to design and validate an online questionnaire to survey relevant and diverse parents’ behaviors associated with key areas for family functioning. Therefore, based on the scientific references about positive parenting and parental support, a set of items capturing commonly represented conceptual dimensions related to parenting were organized into four scales [18,26], explicating the capacities of parents in ensuring basic care and in responding to their children’s developmental needs (daily routines); in promoting stability and family involvement, especially among the main caregivers (co-parenting); in modeling emotional warmth (emotional experiences); and in fostering support for the family via social networks (support network). A fifth and last scale was added for assessing the parents’ emotional mood during the lockdown period, which is out of the scope of the present paper. The items were reviewed and discussed by a group of experts in positive parenting research. The final version of the survey was introduced on an online platform. The online survey also included a set of questions about sociodemographic characteristics (gender, age, job statuses, number of children, age of children, and number of days in lockdown).

Confirmatory factor analysis (CFA) used in the four parental scales is detailed according to goodness-of-fit statistics, respectively the fit indices of chi-square (χ^2^), the comparative fit index (CFI), the Tucker-Lewis index (TLI), and the root mean square error of approximation (RMSEA):

- Daily routines. This scale assembles 15 items related to basic care in daily chores (hygiene, meals, sleep hours, shared and nonshared activities, limits, and guidance), distributed among four subscales: positive discipline (e.g., item 3, “I maintain my children’s wake times and bedtimes during school days”); parent-child activities (e.g., item 8, “I am available to supervise my children’s school work”); nurturing (e.g., item 2, “I make my children take responsibility and help out with household chores”); and enriched environment (e.g., item 15, “I plan at least one different activity for the weekend”). The internal consistency coefficient for this sample was 0.81. The confirmatory factor analysis for the daily routine scale showed satisfactory indices (X^2^ (84) = 138,422 (*p* < 0.001), CFI = 0.957, TLI = 0.947 and RMSEA = 0.044).
- Co-parenting. This scale assembles 11 items assessing marital involvement and mutual support in multiple childcare duties. The items were distributed among two subscales: parental alliance (e.g., item 1, “We decide how to solve our children’s discipline problems together”) and parental agreement (e.g., item 11, “We point out each other’s good qualities to our children”). The internal consistency coefficient for this sample was 87. The confirmatory factor analysis for the co-parenting scale showed satisfactory indices (X^2^ (42) = 233,041 (*p* < 0.001), CFI = 0.922, TLI = 0.898 and RMSEA = 0.099).
- Emotional experience. This scale assembles 14 items tapping into emotional expression. The items were distributed into four subscales: emotional sensitivity (e.g., item 2, “I pay attention to what my children say and how they feel”); emotional tension (e.g., item 7, “I recognize that it does not take much for me to run out of patience”); emotional regulation (e.g., item 6, “I have had to be extra patient when taking care of several things at the same time”); and stress management (e.g., item 9, “I try to control myself so I do not pass on my worries to my children”). The internal consistency coefficient for this sample was 87. The confirmatory factor analysis for the emotional experience scale showed satisfactory indices (X^2^ (113) = 234,756 (*p* < 0.001), CFI = 0.952, TLI = 0.942 and RMSEA = 0.059).
- Support network. This scale included nine items evaluating the changes in the support of formal and informal networks provided to the family during lockdown. The items were distributed into two subscales: informal network (e.g., item 1, “I feel like my family and friends care about my children”) and formal network (item 5, “I sense that educators/teachers care whether my children learn”). The internal consistency coefficient for this sample was 82. The confirmatory factor analysis for the support network scale showed satisfactory indices (X^2^ (25) = 52,521 (*p* < 0.001), CFI = 0.979, TLI = 0.969 and RMSEA = 0.061).

The responses of the four scales were rated on a 5-point scale ranging from 1–5, reflecting the degree of change (1 for “happens much less often”, 2 for “happens less often, 3 for “happens the same”, 4 for “happens more often”, and 5 for “happens much more often”). A score of zero was included to allow the participants to check whenever the item(s) did not apply.

A last open question was included in the survey: “Would you like to mention any situation that has impacted you and your family during the lockdown?”

### 2.4. Procedure

The survey was introduced on an online platform. Before answering the online survey, each participant acknowledged the aims and procedures of the study, including assurance of confidentiality and informed consent. The instructions reminded participants to focus on the lockdown period when responding to the items.

The online survey was available on Facebook, WhatsApp, and other social media platforms from 15 May–26 June, the last day of the school year and end of classes.

Data analysis was carried out using SPSS, version 27, for Windows (IBM Corp. Released 2020. IBM SPSS Statistics for Windows, Version 27.0. Armonk, NY, USA: IBM Corp). Descriptive analyses were performed for all of the dependent variables (the results are provided in the four scales and respective subscales) and one-sample t-tests were used to determine the differences between the scales and the subscales and the mid-point response that indicates no change. A sociodemographic variable was created regarding the parents’ employment statuses (independent variable). Four groups were identified: at-home parents with children under 12 with a licensed work leave and governmental aids (group A), at-home parents who were teleworking (group B), parents working out-of-home as usual (group C), and parents who were unemployed (group D). After checking all of the statistical assumptions, one-way ANOVAs were performed to analyze the difference between means (differences in parenting dimensions according to the parent’s group), and post hoc ANOVAs (Bonferroni test) were performed (with Bonferroni adjustment) to compare the groups for the total score for the scale and its four subscales.

The answers to the open questions were analyzed with QSR N’Vivo QSR (International Pty Ltd. (2018) NVivo (Version 12), Burlington, MA, USA) for Mac.

The parents’ written narratives were first analyzed and coded by the third researcher using an inductive process to explore the data and to organize the emergent categories and subcategories [35]. After that, a discussion was conducted, between three researchers, about the emergent categories and the categories’ taxonomy and, after reaching consensus, these were organized along three thematic axes and nine categories. Finally, we adopted a concurrent triangulation strategy [26] between the emergent categorization and the dimensions of the four parenting scales.

The study was approved by the Ethics Commission for Research in Social and Human Sciences of the University of Minho (reference CEICSH045/2020 of 13 May 2020).

## 3. Results

In this section, we present the quantitative results (descriptive statistics and analyses of variance for the scales and its subscales according to the parents’ employment statuses and the Post hoc ANOVA (Bonferroni test) for differences between groups for the total scale score and its four subscales (Bonferroni correction was applied and the value of *p* < 0.08 was considered for statistical significance), the qualitative results (categories of the content analysis), and the concurrent embedded strategy.

### 3.1. Results for Parenting Dimensions

Table 1 presents the descriptive statistics of the scales and subscales related to changes in parenting behaviors. Measures of central tendency (mode, median, and mean) were computed to summarize the data on the parenting variables. Measures of dispersion (standard deviation) were computed to understand the variability of the scores across these variables. The following are the results of this analysis. The majority of parents did not report changes in how they parented during the lockdown period studied. This trend is clear when taking into account that the most frequent response lies in point 3 (mode = 3, “happens the same”). An exception to this trend is seen for stress management items where the mode lies one point above, indicating more frequent changes (mode = 4; “happens more often”). Nonetheless, for some scales and subscales, the median is higher than 3 (daily routine, parent-child activities, nurturing, emotional experience, emotional sensitivity, emotional tension, emotional regulation, and stress management). The mean scores for all variables are between 3–4 (ranges from 3.05 in parental alliance to 4.04 in stress management), indicating that the mean for the responses of participants tends to be between “happens the same” and “happens more often”. However, the variation range for the scores of each scale and subscale reveals results from 1 (“happens much less often”) to 5 (“happens much more”), reflecting that some parents felt changes in quite different ways (from much less to much more often behaviors), albeit the mean scores of the parents’ answers being between no behavior changes and happens more often.

The one-sample *t*-test shows that the mean values of the subscales presented statistically significant differences, hence leading us to reject the null hypothesis (all population means equal 3). The greatest deviations from the value of reference are reported across the confidence intervals, respectively, on the scale emotional experience (*t* (1257) = 9190, *p* < 0.001, *d* = 0.438, IC = 0.509–0.558), and on the subscales nurturing (*t* (1344) = 35,909, *p* < 0.001, *d* = 0.557, IC = 0.516–0.575) and stress management (*t* (1221) = 46,445, *p* < 0.001, *d* = 0.779, IC = 0.992–1.08).

### 3.2. Analysis of Parenting Changes According to Their Employment Statuses

#### 3.2.1. Daily Routine

Table 2 displays the means and standard deviations for the daily routine scale and its subscales as well as the analyses of variance (one-way ANOVA) for each of the four groups of parents: at-home parents with children under 12 with a licensed work leave and governmental aids (group A), at-home parents who were teleworking (group B), parents working out-of-home as usual (group C), and parents who were unemployed (group D).

As can be seen, the data revealed significant differences between the groups in the total score for the scale and across the four subscales: daily routine total score (F (3, 1378) = 20,467, *p* < 0.001, η^2^ = 0.043); positive discipline (F (3, 1378) = 7.384, *p* < 0.001, η^2^ = 0.016); parent-child activities (F (3, 1368) = 26,514, *p* < 0.05, η^2^ = 0.055); nurturing (F (3, 1341) = 3914, *p* < 0.01, η^2^ = 0.009); and enriched environment (F (3, 1352) = 12,777, *p* < 0.001, η^2^ = 0.028).

The post hoc ANOVA (Bonferroni test) with Bonferroni adjustment (value of *p* < 0.008 was set for statistical significance) showed significant differences between groups for the total score for the scale and its four subscales. At-home parents providing care for their children showed higher levels of statistically significant changes (M = 3.47, SD = 0.451) in the total daily routine score than parents who were teleworking (M = 3.21, SD = 0.456) and parents working out-of-home (M = 3.26, SD = 0.454). For the enriched environment subscale, at-home parents caring for children under showed higher levels of statistically significant changes (M = 3.43, SD = 0.768) than parents who were teleworking (M = 3.13, SD = 0.705), parents working out-of-home (M = 3.16, SD = 0.674), and parents who were unemployed (M = 3.13, SD = 0.756). For the positive discipline subscale, at-home parents providing care for their children and governmental aids showed higher levels of statistically significant change (M = 3.21, SD = 0.553) than at-home parents who were teleworking (M = 3.03, SD = 0.523) and parents working out-of-home (M = 3.07, SD = 0.529). Finally, for the parent-child activities subscale, at-home parents who were teleworking showed a statistically significant reduced change pattern, therefore indicating less parent-child activities (M = 3.35, SD = 0.864) compared with at-home parents providing care for children supported with governmental aids (M = 3.85, SD = 0.700) and parents working out-of-home (M = 3.52, SD = 0.749).

#### 3.2.2. Co-Parenting

Table 3 displays the means and the standard deviations for the co-parenting total scale and its subscales as well as the analyses of variance (one-way ANOVA) for each of the four groups of parents.

As can be seen, the data revealed significant differences between the groups in the total score for the scale and the two subscales: co-parenting total score (F (3, 1235) = 7323, *p* < 0.001, η^2^ = 0.017); parental alliance (F (3, 1226) = 9.782, *p* < 0.001, η^2^ = 0.023) and parental agreement (F (3, 1231) = 4.527, *p* < 0.001, η^2^ = 0.01).

The post hoc ANOVA with Bonferroni adjustment (value of *p* < 0.008 was set for statistical significance) revealed significant differences between groups for the total scale score and its two subscales. At-home parents who were teleworking showed lower levels of statistically significant changes for the co-parenting total score (M = 3.06, SD = 0.463) compared with at-home working parents providing childcare with governmental aids (M = 3.21, SD = 0.493). Finally, regarding the subscale for parental agreement, at-home parents who were teleworking kept the same change pattern, with low levels of statistically significant changes (M = 3.11, SD = 0.517), compared with at-home parents providing care with governmental aids (M = 3.24, SD = 0.546).

#### 3.2.3. Emotional Experience

Table 4 displays the means and standard deviations for the total scores for emotional experience and the subscales as well as the analyses of variance (one-way ANOVA) for the four groups of parents.

As can be seen, the data revealed significant differences between the groups in the total score for the scale and the four subscales: emotional experience (F (3, 1254) = 3017, *p* < 0.05, η^2^ = 0.007); emotional sensitivity (F (3, 1204) = 3.345, *p* < 0.05, η^2^ = 0.043); emotional regulation (F (3, 1245) = 3.486, *p* < 0.05, η^2^ = 0.016); and stress management (F (3, 1218) = 13,967, *p* < 0.001, η^2^ = 0.028). For emotional tension, no significant differences were found between groups (F (3, 1245) = 1637, *p* < 0.05, η^2^ = 0.004).

The post hoc ANOVA (Bonferroni test) with Bonferroni adjustment showed significant differences between the groups for stress management. At-home parents who were teleworking showed higher levels of statistically significant changes, indicating an increased coping effort (M = 4.19, SD = 3.88), compared with at-home working parents providing childcare supported by governmental aids (M = 3.96, SD = 0.823), parents working out-of-home (M = 3.88, SD = 0.784), and parents who were unemployed (M = 3.89, SD = 0.776). The total score for emotional experience did not register significant differences between groups.

#### 3.2.4. Support Network

Table 5 displays the means and standard deviations for the total score for support networks and its subscales as well as the analyses of variance (one-way ANOVA) for the four groups of parents.

As can be seen, the data revealed significant differences between groups in the total score for the scale and the two subscales: support network total score (F (3, 1206) = 3675, *p* < 0.05, η^2^ = 0.009); informal network (F (3, 1151) = 5.05, *p* < 0.05, η^2^ = 0.002) and formal network (F (3, 1204) = 3.3, *p* < 0.05, η^2^ = 0.009).

Again, considering the Bonferroni adjustment, the post hoc ANOVA showed significant differences between the groups for the informal network subscale. At-home parents who were teleworking showed lower levels of statistically significant changes (M = 2.99, SD = 0.663) compared with parents who were unemployed (M = 3.24, SD = 0.076).

### 3.3. Open Questions (Narratives)

Three thematic axes emerged from the answers to the open question in the survey (“Would you like to mention any situation that has been impacting during the time of the family’s lockdown?”). These axes were distributed into family dynamics professional activity and school community’s relationship (Table 6).

The theme family dynamics represents the most prevalent axis. It aggregates five categories, embracing positive and negative connotations. Category one, strengths and opportunities, is the most quoted (138), gathering nearly half of the respondents’ quotes. Most of them (84) perceived the lockdown to be extended time taken for the family members to gather and for parent-child activities. Some parents (54) realized that their coping mechanisms evolved from these parent-child shared activities. Category two, affective and emotional expressions (f = 79), included positive (45) and negative (34) feelings related to the lockdown. Some parents revealed an accrued sensitivity to their children’s emotional needs while others declared that they were overwhelmed with the responsibility and multiplicity of tasks. Category three, weaknesses and threats (f = 40), displays the parents’ references to difficult work-life balances and the disruption in contact with relatives. Category four, marital relationship (f = 11), mirrors the differential impact of reality for parents experiencing the harshness of a divorce during the lockdown, whereas for others, the lockdown re-nurtured marital and family relationships. Category five, family health and bereavement (f = 9), included mainly parents’ quotes on illness, death, and bereavement, pointing to the presence of stressors in their personal lives and for the family’s well-being. The second theme, relationship with the school community, encompasses all opinions referencing school, learning, home schooling, parent–teachers relations, and online socialization. Parents expressed losses (f = 17) through their concerns and feelings of helplessness whenever the child showed higher sensitivity to the change in school activities. In a similar vein, parents underlined gains (f = 5) related to increased participation in their children’s learning activities, and feelings of communion and mutuality in online groups of parents.

The third theme, professional activity, presents the differentiation in parent’s work statuses and its impact on personal and family lives. Opinions on teleworking were expressed by some participants, predominantly mothers expressing losses (f = 9) due to the burden of professional demands, domestic activities, and childcare. Lack of leisure caused feelings of tension and burnout. A minor group of references related gains (f = 3) to governmental aids, a reduction in working hours and better work-life balance. Finally, quotes on the experiences of working out-of-home express worries about the situation, feelings of uncertainty, and how some work conditions were particularly stress-inducing (f = 3). Despite some categories emerging from only a few participants, they are mentioned in Table 6 because they introduced some aspects that impacted families, and according to the study purposes, a wider understanding of family changes is important. In addition, we also think those are aspects that might give some insights on what to focus upon for future studies.

### 3.4. Current Embedded Strategy

**Figure 1:**
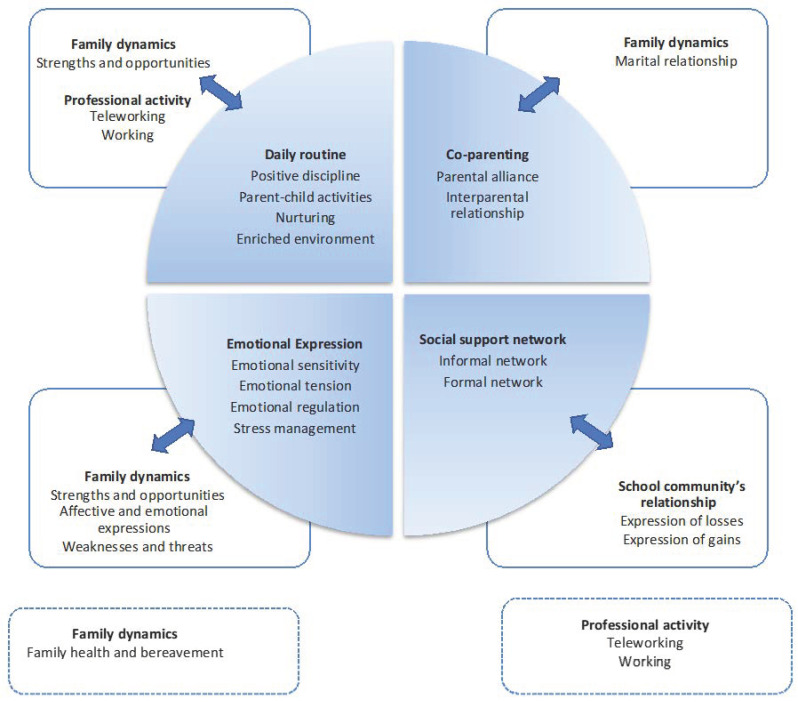
Comparison of the parenting scales and subscales of the survey, and the thematic axes and categories from the content analysis of the responses.

Intersections between the results of the scales and the content analysis of the responses were searched. We used the concurrent embedded strategy [33] to devise comparisons at different levels of the quantitative analysis (results of the scales and subscales) and the qualitative content analysis (themes and categories). In Figure 1, we present the results of this crossover. This qualitative analysis allowed us to extend and add specific aspects to each of the domains surveyed in the parenting COVID-19 questionnaire. A more comprehensive perception about the impact of lockdown on daily family routines, marital relationships, emotional experiences, and support granted from the family’s informal and formal networks was grasped from the parents’ responses to the open questions (see Table 6). In the quotes related to their experiences and how these affected family households, relations, and themselves, parents brought new situations, gave more information on the key areas of family functioning, while supplementing their perspectives and clarifying the emotional tone of the descriptions. For instance, changes in the family dynamics were appraised as strengths and opportunities. Inversely, parents did not elude weaknesses and threats surrounding the abrupt change and the novelty of the situation. In all of the thematic axes, gains and losses were identified as ongoing processes. Additionally, the combination of analyses allowed us to obtain the impact of the circumstances and its meaning considering each family context. Globally, this mixed strategy tapped into the quantitative and qualitative dimensions. Worth mentioning is that some new topics emerged from the narratives, though for only a few participants. Those were related to family dynamics, such as care for those who are older, illness, death, and bereavement, and to professional activities, namely, teleworking and working constraints.

## 4. Discussion

In this study, we examined how the parents responded to changes during the COVID-19 lockdown throughout two different sets of data performing both quantitative and qualitative analyses. Outstandingly, the parents reported a high degree of stability along daily routines, co-parenting tasks, and networking with their informal and formal groups of support. Over and above the stable occurrence of habits, customs, and parental behaviors, the parents showed increased susceptibility to stress-inducing situations, which demanded a surplus of effort to manage and control emotions.

Indeed, a first remark is for the diversity of family experiences and the myriad of meanings attached to the impact of the COVID-19 lockdown. True, some families highlighted the time spent with their family, the opportunity for providing better childcare and monitoring their children’s activities, the benefits of teleworking, and the ability to adjust to a new situation. However, none of the parents’ appraisals regarding the gains eluded some emotional turmoil, since stress levels were higher. In fact, this corroborates official surveys reporting the employees’ perspective concerning working from home to be a far more demanding experience than earlier expected. Still, other parents were appreciative of “the absence of time and stress of commuting to the office; spending more time with their children and spouses; and also, the flexibility of the working hours” [9] p. 17.

Examining the four groups of parents identified according to different employment statuses (at-home working parents providing care for their children with governmental aids, parents teleworking at home, parents working out-of-home, and parents who were unemployed), we perceived changes in key areas of parenting associated with these statuses, as detailed below:

- Parents working at home providing care for their children

Globally, the parents of children needing care who were home on governmental aids revealed more positive changes in daily routine activities when compared with the other groups of parents, namely, associated with the use of positive discipline and an enriched environment. Besides, drawing from the coparenting dimension and, particularly, looking at the levels of parental agreement, this group of parents is better off than his congener teleworking at home. In addition, the same can be said when comparing the emotional experience of these two groups of parents, since parents who were home on governmental aids showed to be spared from the charge of parents teleworking at home. These results allow us to think that this licensed parental leave was quite beneficial for family routines and for better psychological adjustment, which comprehensively allowed them to benefit from their new normal and to ease their parental role. Similar to families in other countries, this group of Portuguese families [9] also perceived gains from this governmental measure. Reasonably, the parents could enjoy their parenting role while educating and supporting their children in play or school activities and other demands. Illustrating the tenacity of parents to cope with the lockdown and to search for positive solutions, time spent at home was consistently quoted as a gain for the family dynamics and the enhancement of strengths and opportunities. In line with Froma Walsh’s conceptualization of family resilience, this atypical pandemic event elicited opportunities to develop family creativity, problem resolution, and sustained perseverance [17]. Suffice to say that, once these internal resources of family resilience enhance ways to mitigate or even alleviate the stress, at the same time, they operate levers for a better psychological adjustment and break even the adverse costs of the pandemic restrictions.

- Parents teleworking at home

Distinctively, parents teleworking perceived themselves as being less active in parent-child activities and less backed up from their partners, as shown in the lower levels of parental agreement when compared with the group of working parents on governmental support to provide child care. On the other hand, parents working remotely from home indicated higher results in stress management than other groups of parents. Yet what regards the support network, more worryingly, is the reported change in the most proximal and affectionate circle of support rendering visible the distress of parents who were teleworking at home. For this group, the informal network comes out more weakened than amongst the group of parents at home providing childcare on governmental aids or the parents who were unemployed. These results allow us to conclude that this group of Portuguese parents felt more constraints to engage in activities with their children such as playtime or school tasks. In the same vein, they perceived their marital relationship to affect and be affected by less agreement. Not without mentioning, support from the informal network falls shorter than usual or undergoes difficulties. Research on teleworking suggests that, even when children are not at home, parents continue to experience work overload and stress from the work-family connection. There are advantages such as better control over working hours, but this does not prevent the stress accumulating from the relationship between care provision, domestic work (including the management of children’s school and leisure activities), and work activities [36]. In the literature related to the COVID-19 pandemic, numerous consequences are documented, particularly changes and problems in the couple’s relationship and work [12]. Additionally, it proved to be a tough challenge for parents working from home to balance work and childcare when schools and other care institutions were closed. Consequently, their children were at home at the same time that they were working [9]. In addition, parents who were remote workers showed higher changes in stress management, indicating that these parents made a bigger effort to respond to their children’s needs and their jobs. Nonetheless, their involvement in parent-child activities was less and co-parenting was not totally satisfying, maybe aggravated due to an awareness of the lacking number of activities they engaged in with their children, the lack of time for themselves, difficulty in their work-life balance, and feelings of exhaustion. Therefore, teleworking during COVID-19 required parents to articulate between work demands, parenting, and school support for their children. This multiplicity of roles seems to demand more from parents, making feeling competent and satisfied in the different areas more difficult. This may result in higher levels of stress and anxiety [37].

Thus, corroborating the demands of a work-life balance, parental stress, and feelings of exhaustion might also explain our results since other studies revealed depressive, anxious, and stressful symptomatology among parents [6,8,11]. Congruent with current reports on teleworking during COVID-19 [9], in the present study, we reiterate the difficulty of maintaining a work-life balance. In addition, the confinement demands are further complicated if at-home parents who were teleworking are “single parents or parents of children with a disability or learning difficulty” [9] p. 17.

- Parents working out-of-home

The group of parents working out-of-home kept their usual job placements. Along with different parenting dimensions, the pattern of change in this group was not particularly expressive neither in itself nor in comparison with the three other groups. Across the parenting dimensions studied (daily routines, co-parenting, emotional experiences, and support networks), the changes in this group were residual when considering the central tendency measures. In parallel, parents working out-of-home did not increase their quality of parenting the way that the group of parents who benefitted from a governmental paid leave to stay home in childcare did. However, when compared with the group of parents in telework or the parents who were unemployed, parents working out-of-home were not as hampered, especially when comparing the loss of formal networks for the group of parents who were unemployed. Despite parents working out-of-home having to worry about health security measures, they did not manifest higher stress levels or the intense emotional experiences of at-home parents in telework.

- Parents who were unemployed

The group of parents who were unemployed are not particular visible in the group comparisons. However, across the parenting dimensions under study, the most affected one seems linked to the support of the formal network. Although not reaching statistically significance, the lowest score on this dimension sets a sign of worry. As rated and echoed in the open-ended answer, this group of parents felt that, during lockdown, the formal structures of support were not available or capable of appeasing their needs. This is a concerning point because this group of parents and their families were already a vulnerable group and, seemingly, the lockdown further weakened their support networks. Indeed, the consequences of the COVID-19 pandemic should not be regarded as a health issue spilt over to other important life conditions [7]. Moreover, the support of schools is crucial [16] and parents who are deprived from that support tend to experience more difficulties. Apart from this vulnerability, the group of parents who were unemployed did not evidence any other concerning psychosocial impairments.

Summing up these analyses on the parents’ differing working statuses, we mention that they allowed us to sort out the influence of work conditions in parenting and the family context during this global pandemic. We note that, importantly, the changes in emotional experiences were transversal to all groups of parents and that neither of them differed from each other concerning the level of emotional tension. These results are congruent with results from other studies that evidence that confinement periods could lead to psychological problems and stress [5,6,8,11]. In fact, psychological distress among parents was recently documented in the literature as a consequence of COVID-19 lockdowns [6].

How each family experienced the pandemic situation seems to be influenced by multiple factors. Socioeconomic status seems to determine how parents adjusted to professional demands and how they invested in their parental role. Therefore, considering that the well-being of families during this period could be better explained by considering their different adversities, traumas, stresses, as well as available resources and support is important [38]. Despite current national and international difficulties, research cannot stop, namely those related to the social implications of the COVID-19 pandemic on families. To this purpose, we purposely intended to report on a mixed-methods study, which enabled us to acquire comprehensive knowledge on this new and tempestuous subject. Overall, the present study brings some new perspectives on the efforts and strengths of Portuguese families in coping with the COVID-19 confinement, where government support and teleworking seemed to influence work and family lives globally as well as the well-being of Portuguese families.

The use of a mixed methodology allowed us to evidence how the two methods of analysis extended and contributed to a better understanding of family experiences during this atypical time. The quantitative data allowed us to analyze the patterns in the changes that occurred in the abovementioned key areas for family functioning while the qualitative analysis tapped in the subjectivity of individual experiences. The combination of data collected showed that the lockdown is associated with both appealing and parent-fulfilling experiences and, at the same time, challenging and stress-inducing events.

Nonetheless, some limitations were present in the results of this study. The use of a self-report measure can be biased to subjective appraisals and particularly sensitive to individual mood variations during extended COVID-19 times. Our ad hoc questionnaire emphasized the support from schools and was not so specific about other sources of formal support; therefore, the data on support networks should be carefully interpreted. Further limitations of the study can be reported: the online collection data procedure lacks representativeness, favoring participants with similar SES characteristics and/or higher digital literacy levels, who are more likely to have access. Additionally, the number of at-risk families was not evident in the study. A bias towards the participation of mothers was registered, since fewer fathers participated.

Finally, an interesting observation is the better-off position of at-home parents with a special work-paid leave to take care of children under twelve years old. The governmental financial support for families was seemingly regarded as a good investment and surely a message recognizing parents on behalf of all children with the right to be parented against all odds.

## Figures and Tables

**Table 1:** Descriptive statistics of scales and subscales related to parenting behaviors and one sample *t*-test.

Scale and Subscales	Mode	Mdn	M(SD)	Range	*t*	*p*	*d*	IC 95%
**Daily Routine**	3	3.21	3.29 (0.463)	1.38–5	23.089	<0.001	0.463	(0.263–0.312)
Positive discipline	3	3	3.09 (0.534)	1.5–5	5.995	<0.001	0.534	(0.058–0.114)
Parent-child activities	3	3.67	3.52 (0.810)	1–5	23.925	<0.001	0.810	(0.480–0.566)
Nurturing	3	3.5	3.55 (0.557)	1.5–5	35.909	<0.001	0.557	(0.516–0.575)
Enriched environment	3	3	3.20 (0.722)	1–5	10.061	<0.001	0.722	(0.159–0.236)
**Co-parenting**	3	3	3.12 (0.475)	1–5	9.190	<0.001	0.475	(0.098–0.151)
Parental alliance	3	3	3.05 (0.482)	1–5	2.748	0.006	0.482	(0.011–0.068)
Parental agreement	3	3	3.17 (0.528)	1–5	11.682	<0.001	0.528	(0.146–0.205)
**Emotional Experience**	3	3.5	3.53 (0.438)	1.75–5	43.194	<0.001	0.438	(0.509–0.558)
Emotional sensitivity	3	3.2	3.44 (0.583)	1–5	26.072	<0.001	0.584	(0.405–0.471)
Emotional tension	3	3.5	3.52 (0.711)	1–5	25.985	<0.001	0.711	(0.484–0.563)
Emotional regulation	3	3.2	3.32 (0.562)	1–5	20.340	<0.001	0.563	(0.293–0.356)
Stress management	4	4	4.04 (0.779)	1–5	46.445	<0.001	0.779	(0.992–1.08)
**Support network**	3	3	3.06 (0.603)	1–5	3.648	<0.001	0.603	(0.029–0.097)
Informal network	3	3	3.04 (0.687)	1–5	2.144	0.032	0.686	(0.004–0.081)
Formal network	3	3	3.06 (0.751)	1–5	2.782	0.005	0.751	(0.018–0.105)

**Table 2:** Descriptive statistics and analyses of variance for the daily routine scale and its subscale according to the parents’ employment statuses.

	A		B		C		D		ANOVA			Post Hoc
Group	N	M (SD)	N	M (SD)	N	M (SD)	N	M (SD)	F	*p*	η^2^	Bonferroni
Scale and Subscales												
Daily routine	284	3.47	594	3.21	401	3.26	103	3.31	20,467	0.000	0.043	A > B,C *
		(0.451)		(0.456)		(0.454)		(0.444)				
Positive discipline	284	3.21	594	3.03	401	3.07	103	3.12	7384	0.000	0.016	A > B,C *
		(0.553)		(0.523)		(0.529)		(0.518)				
Parent-child activities	284	3.85	592	3.35	395	3.52	103	3.64	26,514	0.000	0.055	A > B,C * B < D *
		(0.700)		(0.864)		(0.749)		(0.700)				
Nurturing	275	3.64	587	3.54	383	3.52	100	3.47	3914	0.009	0.009	ns *
		0.600		(0.540)		(0.552)		(0.528)				
Enriched environment	274	3.43	592	3.13	392	3.16	98	3.13	12,777	0.000	0.028	A > B,C,D *
		(0.768)		(0.705)		(0.674)		(0.756)				

Note: group A = at-home parents with children under 12 with a licensed work leave and governmental aids; group B = at-home parents who were teleworking; group C = parents working out-of-home as usual; group D = parents who were unemployed; * *p* < 0.008 (Bonferroni adjusted *p* value).

**Table 3:** Descriptive statistics and analyses of variance for the co-parenting scale and its subscales according to the parents’ employment statuses.

	A		B		C		D		ANOVA			Post Hoc
Group	N	M (SD)	N	M (SD)	N	M (SD)	N	M (SD)	F	*p*	η^2^	Bonferroni
Scale and Subscales												
Co-parenting	251	3.21	539	3.06	362	3.15	87	3.20	7.323	0.0000	0.017	B < A *
		(0.493)		(0.463)		(0.475)		(0.445)				
Parental alliance	250	3.14	533	2.96	361	3.06	86	3.14	9.782	0.004	0.023	ns *
		(0.505)		(0.473)		(0.477)		(0.045)				
Parental agreement	251	3.24	361	3.11	361	3.21	87	3.23	4.527	0.0000	0.011	B < A *
		(0.546)		(0.517)		(0.527)		(0.506)				

Note: group A = at-home parents with children under 12 with a licensed work leave and governmental aids; group B = at-home parents who were teleworking; group C = parents working out-of-home as usual; group D = parents who were unemployed; * *p* < 0.008 (Bonferroni adjusted *p* value).

**Table 4:** Descriptive statistics and analyses of variance for the emotional experience scale and its subscales according to the parents’ employment statuses.

	A		B		C		D		ANOVA			Post Hoc
Group	N	M (SD)	N	M (SD)	N	M (SD)	N	M (SD)	F	*p*	η^2^	Bonferroni
Scale and Subscales												
Emotinal experience	251	3.57	551	3.56	362	3.49	94	3.46	3017	0.029	0.007	ns *
		(0.480)		(0.419)		(0.429)		(0.451)				
Emotional sensitivity	242	3.54	540	3.39	340	3.43	86	3.46	3345	0.019	0.008	ns *
		(0.568)		(0.581)		(0.545)		(0.545)				
Emotional tension	249	3.50	356	3.57	356	3.49	94	3.44	1637	0.179	0.004	ns *
		(0.764)		(0.699)		(0.682)		(0.739)				
Emotional regulation	250	3.42	550	3.29	356	3.30	93	3.36	3486	0.015	0.008	ns *
		(0.578)		(0.577)		(0.531)		(0.543)				
Stress managment	244	3.96	545	4.19	347	3.88	86	3.89	13,967	0.000	0.033	B > A,C,D *
		(0.823)		(3.88)		(0.784)		(0.776)				

Note: group A = at-home parents with children under 12 with a licensed work leave and governmental aids; group B = at-home parents who were teleworking; group C = parents working out-of-home as usual; group D = parents who were unemployed; * *p* < 0.008 (Bonferroni adjusted *p* value).

**Table 5:** Descriptive statistics and analyses of variance for the support network scale and its subscales according to the parents’ employment statuses.

	A		B		C		D		ANOVA			Post Hoc
Group	N	M (SD)	N	M (SD)	N	M (SD)	N	M (SD)	F	*p*	η^2^	Bonferroni
Scale and Subscales												
Support network	245	3.17	532	3.03	344	3.03	89	3.12	3.675	0.012	0.009	ns *
		(0.622)		(0.588)		(0.593)		(0.650)				
Informal network	243	3.12	532	2.99	344	3.03	89	3.24	5.053	0.02	0.012	B < A,D *
		(0.697)		(0.663)		(0.694)		(0.076)				
Formal network	237	3.18	318	3.06	318	3.00	82	2.93	3.3	0.002	0.009	ns *
		(0.781)		(0.733)		(0.735)		(0.807)				

Note: group A = at-home parents with children under 12 with a licensed work leave and governmental aids; group B = at-home parents who were teleworking; group C = parents working out-of-home as usual; group D = parents who were unemployed; * *p* < 0.008 (Bonferroni adjusted *p* value).

**Table 6:** Thematic axes, categories, and subcategories generated from the narrative responses.

Thematic Axes	Categories (f)	Subcategories (f)	Narrative Excerpts *
Family dynamics	Strengths and opportunities (138)	Extended family time (84)	Longer socialization time among all members of the family. (P15)
		Coping mechanisms (54)	Cooking with my daughter. (P954)
	Affective and emotional expressions (79)	Positive (45)	I believe that, for my daughter, this was a period of affection and comfort. (P286)
		Negative (34)	Less patience, much more to do. (P71)
	Weaknesses and threats (40)	Balancing professional activity, parenting, and domestic life (21)	Overload from responsibilities, housekeeping, and education. (P473)
		Social distancing of family members (19)	My children did not have normal contact with their grandparents. (P901)
	Marital relationship (11)	Expression of losses (7)	Divorce needed. (P226)
		Expression of gains (4)	The marital relationship became more united. I sincerely think that the time spent together has been very positive. (P77)
	Family health and bereavement (9)	Illness in family members (4)	The possibility of a cancer diagnosis in a close family member. (P351)
		Death of a family member (4)	Death of a relative and its funeral restricted to the family. (P30)
		Caregiving for a family member who is older (1)	Living with a person who is older was not always easy. (P31)
Relationship with the School Community	Expression of losses (17)	−	My daughter who has Asperger’s modified her behavior, being extremely resistant to change and to distance teaching. (P515)
	Expression of gains (5)	−	Increased participation and inter-help by other parents of (school) children and internet groups. (P85)
Professional activity	Teleworking (12)	Expression of losses (9)	The entry into teleworking “suddenly” led, so far, to an excess amount of work.… I have the feeling that I’m always working (profession/domestic) and I never have real leisure.… (P547)
		Expression of gains (3)	Due to the fact that I was laied off, teleworking with reduced hours has promoted a greater balance between professional and personal life. (P1208)
	Working (10)	−	My situation is not easy because of the state we are in, and we are out of work because what I earn is barely enough to pay the expenses. (P1203)

f = Frequency of categories and subcategories; * Narrative excerpts were translated to English from the original writing in Portuguese.

## Data Availability

The data presented in this study are available upon request from the corresponding author.
